# Hypergravity down-regulates c-fos gene expression via ROCK/Rho-GTP and the PI3K signaling pathway in murine ATDC5 chondroprogenitor cells

**DOI:** 10.1371/journal.pone.0185394

**Published:** 2017-09-27

**Authors:** Jeonghyun Kim, Kevin Montagne, Hidetoshi Nemoto, Takashi Ushida, Katsuko S. Furukawa

**Affiliations:** 1 Department of Mechanical Engineering, Graduate School of Engineering, University of Tokyo, Tokyo, Japan; 2 Division of Regenerative Medical Engineering, Center for Disease Biology and Integrative Medicine, School of Medicine, University of Tokyo, Tokyo, Japan; 3 Department of Bioengineering, Graduate school of engineering, University of Tokyo, Tokyo, Japan; University of Umeå, SWEDEN

## Abstract

Chondrocytes are known to be physiologically loaded with diverse physical factors such as compressive stress, shear stress and hydrostatic pressure. Although the effects of those mechanical stimuli onto various cell models have been widely studied, those of hypergravity have not yet been revealed clearly. Hereby, we hypothesized that the hypergravity affects relative positions of intracellular elements including nucleus and cytoskeletons due to their density differences, triggering mechanotransduction in the cell. The aim of this study was to investigate the effect of hypergravity on c-fos expression in the murine ATDC5 chondroprogenitor cells, as c-fos is a well known key regulator of cell proliferation and differentiation, including in chondrocytes. We first found that hypergravity down-regulated c-fos expression transiently via ROCK/Rho-GTP and PI3K signaling, and the down-regulation was suppressed by inhibition of actin polymerization.

## Introduction

Along with biochemical stimuli, mechanical stimuli are currently known to trigger essential intracellular signals in various cell species. Mechanical stimuli such as hydrostatic pressure, compressive stress and tensile stress are widely utilized in the field of mechanobiology [1–5]. Among mechanical stimuli, altered gravity involving microgravity and hypergravity has attracted interest for studying the effects of space flight. Since the cytosol and intracellular micro-organelles such as the nucleus, mitochondria, the cytoskeletons including actin fibers, intermediate filaments, and microtubules have different densities, changes in their relative position could result from altered gravity. Several reports have shown that altered gravity exerts various effects on mammalian cell models[6–9]. Generally, it is known that microgravity strongly suppresses bone mass after a long period of exposure[10]. Similarly, effects of altered gravity on articular cartilage could also be significant. While the hypergravity has the advantage of being easily realized *in vitro* by centrifugation, only limited studies are currently available with regard to hypergravity and chondrocytes. In those studies, chondrocytes were subjected to a moderate intensity of hypergravity, namely 1.8 G[11,12], and no studies have discussed chondrocyte behavior under stronger hypergravity. Hence, we hypothesized that the strong hypergravity can trigger mechanotranduction in the cells due to their different densities of the intracellular elements. In this study, we investigated the effects of greater hypergravity on the murine ATDC5 chondroprogenitor cells.

c-Fos and c-Jun are part of a family of proteins that dimerize to form the transcription factor AP-1, known to regulate the expression of target genes involved in cell division and differentiation[13,14]. For example, AP-1 regulates the gene expression of matrix metalloproteinase (MMP) family members, which play a critical role in the pathogenesis of osteoarthritis (OA), showing the importance of carefully monitoring c-fos expression[15]. It is also known that the overexpression of c-fos suppresses chondrocyte differentiation[16]. Moreover, c-fos is regarded as an early response gene to both biochemical and mechanical stress in various cell types[17–23].

The aim of this study is to investigate the effect of hypergravity in murine ATDC5 chondroprogenitor cell, focusing on the c-fos gene. In this study, we first carried out a 3 day-experiment by applying hypergravity to ATDC5 monolayers with centrifugation, followed by a shorter time-course experiment (0, 30, 60, and 120 min). Then, a dose-response experiment (1, 18.7, 33.3, 52.0, 207.9, and 467.9 G) was performed based on the result from the time-course experiment to identify the most effective hypergravity intensity in terms of c-fos gene modulation. In order to investigate upstream mechanisms of this effect precisely, we focused on actin polymerization using an inhibitor of actin polymerization, and downstream pathways, using a ROCK inhibitor and a PI3K inhibitor. Cytochalasin D was used as an inhibitor of actin polymerization to check any changes in actin cytoskeleton under hypergravity since the cytoskeleton has been reported to be changed due to excessive gravity[7]. Moreover, another study showed that ROCK overexpression increased the transcriptional activity of c-fos in NIH 3T3 cells[24]. Y27632 was employed to inhibit the activation of ROCK, located downstream of the Rho family, which plays a key role in actin cytoskeleton reorganization[24–27]. Through those inhibitor experiments, we tried to investigate possible signaling pathways triggered by hypergravity, resulting in down-regulation of c-fos expression in the ATDC5 cells.

## Materials and methods

### ATDC5 cell culture

The murine chondroprogenitor cell line ATDC5 cells were purchased from the Japanese Collection of Research Bioresources Cell Bank. The ATDC5 cells have been widely utilized for *in vitro* study on chondrocytes[28–30]. We cultured ATDC5 cells in high-glucose Dulbecco’s modified Eagle’s medium (GIBCO)/Ham’s F12 (GIBCO) (1:1), supplemented with 5% of fetal bovine serum (GIBCO) and 1% of Antibiotic-Antimycotic solution (GIBCO) in a humidified incubator at 37°C with 5% CO_2_. When making monolayer samples, 2.0 × 10^5^ cells were seeded onto 35mm Petri dishes. After 4 days of culture in the incubator, confluent ATDC5 cell monolayers were formed.

### Hypergravity conditions

ATDC5 cells were cultured in the incubator at 37°C for 4 days to form monolayers and then exposed to hypergavity for a certain time using a centrifuge (Himac CF7D2; Hitachi). The gravity loaded was calculated as shown in the following equation; Gravity = 1118 × (radius) × (RPM)^2^ × 10^−8^. Control samples were also prepared and kept in the same conditions without hypergravity.

We first loaded 32 G of hypergravity to the ATDC5 monolayers 1 hour daily for 3 days. Since c-fos is known to be an early response gene to biochemical and mechanical stimuli, we first performed a time-course experiment for short periods (0, 30, 60, and 120 min) of exposure to hypergravity, followed by a dose-response experiment by modulating the intensity of hypergravity (18.7, 33.3, 52.0, 207.9, and 467.9 G). Based on the results of both the time-course and dose-response experiments, inhibitor tests were carried out for real-time PCR, staining and western blotting.

### Real-time PCR

In this study, we performed real-time PCR (rt-PCR) in order to measure changes in mRNA expression under hypergravity. After centrifugation, samples were immediately collected and lysed in Trizol reagent (Invitrogen). Then, RNA was extracted and cDNA synthesized from 500 ng of total RNA using ReverTra Ace^®^ qPCR RT Master Mix with gDNA Remover (TOYOBO). In order to examine the effect of hypergravity in ATDC5 cells, we measured the mRNA expressions of c-fos and c-jun. The mRNA expressions were normalized to Glyceraldehyde-3-phophate dehydrogenase (*Gapdh*) and Ribosomal protein L13a (*Rpl13a*) expression. The sets of gene-specific oligonucleotide primers are as follows; *Gapdh*: forward 5’-AAATGGTGAAGGTCGGTGTG-3’, reverse 5’-TGAAGGGGTCGTTGATGG-3’, amplicon size 108 bp; *Rpl13a*: 5’-TCTGGAGGAGAAACGGAAGGA-3’, reverse 5’-GGTTCACACCAAGAGTCCATTG-3’, amplicon size 151 bp; *Fos*: 5’-CACTCCAAGCGGAGACAGAT-3’, reverse 5’-GGCTGCCAAAATAAACTCCA-3’, amplicon size 107 bp; *Jun*: 5’-ATGGGCACATCACCACTACA-3’, reverse 5’-GACACTGGGAAGCGTGTTCT-3’, amplicon size 137 bp.

### Staining

After loading hypergravity, the cell monolayers were treated with 4% paraformaldehyde (PFA) to fix the cells. Then, staining of the nuclei (using DAPI; Thermo Fisher Scientific) and acting filaments (using Actin-stain^™^ 555 Fluorescent Phalloidin; Cytoskeleton, Inc.) was carried out.

### Western blotting

Immediately after loading hypergravity, the cells were lyzed in RIPA buffer containing 0.5% phosphate inhibitor cocktail 3 (Sigma), 1mM EDTA, 1mM NaF, and 2mM Na_3_VO_4_. The lysed cells were then centrifuged at 12,000 rpm for 5 min at 4°C, and the supernatants were mixed with loading buffer 2% SDS, 1M DTT, and Laemmli sample buffer (Bio-Rad)). The total protein quantification was carried out using the Bio-Rad DC protein assay kit (Bio-Rad). Based on the results of the protein quantification, equal amounts of protein in each sample were separated in a polyacrylamide gel (10% Acrylamide, 390mM Tris HCl (pH8.8), 0.05% SDS, milli-Q water, 0.04% TEMED, and 0.1% APS) by SDS-PAGE and transferred to PVDF membranes (Bio-Rad). After blocking the membranes with 5% non-fat milk in Tris Buffered Saline with Tween-20 (TBST) for 1 h at room temperature, the membranes were incubated with the primary antibody for 1 h at room temperature or overnight at 4°C. For visualizing the primary antibody, the membranes were incubated with an HRP-conjugated goat anti-rabbit IgG antibody in TBST for 1 h at room temperature. Protein bands were revealed by enhanced chemiluminescence (ECL, Roche) and imaged using a LAS-3000 imaging system (Fujifilm).

### Statistical analysis

All bars of rt-PCR data represent the means ± standard error. A Student’s t-test was performed to calculate p-values in order to assess the statistical significance of the observed differences.

## Results

### Hypergravity significantly down-regulated c-fos expression

**Fig 1:**
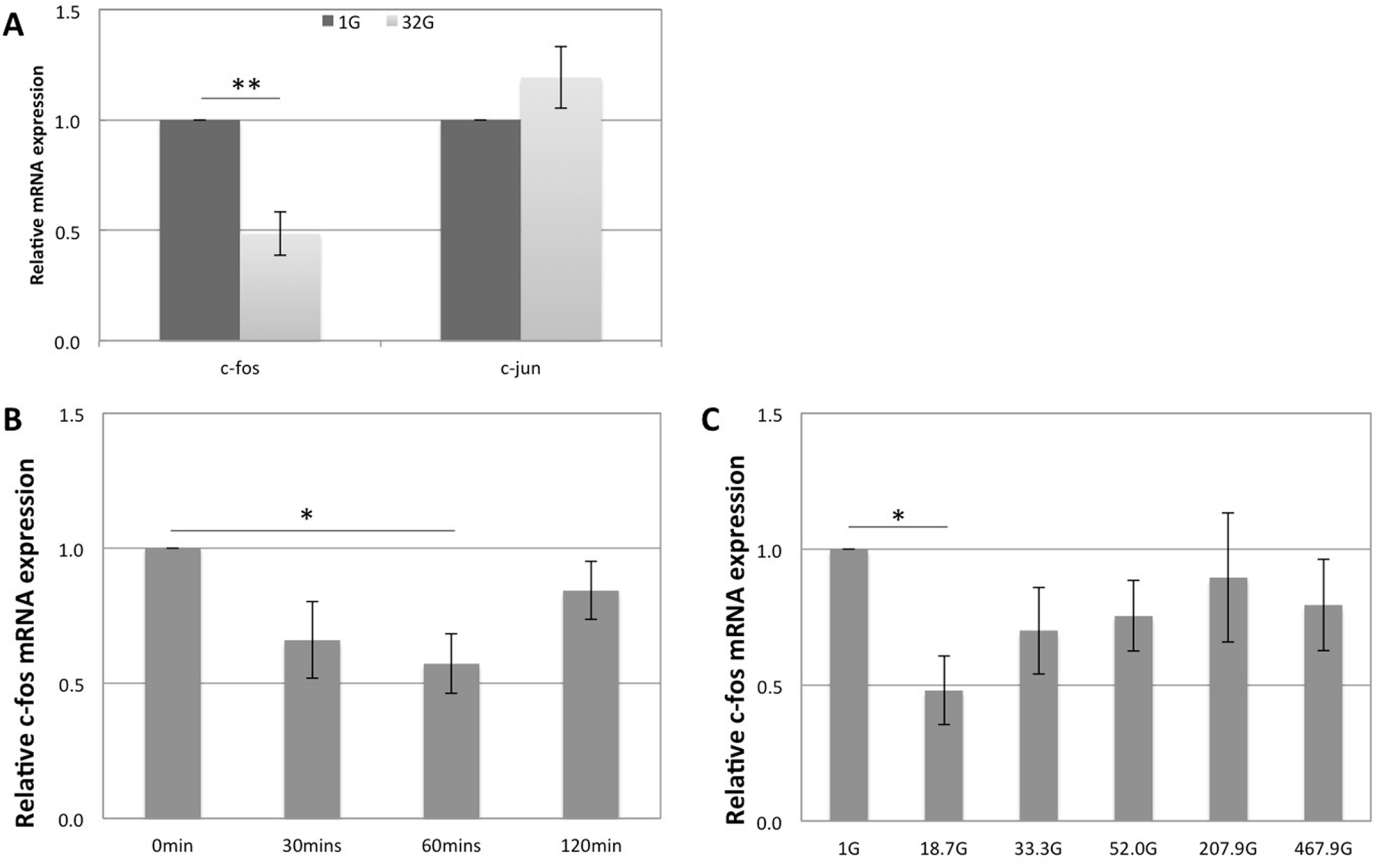
Hypergravity down-regulated c-fos expression in ATDC5 cells. (A) mRNA expression of c-fos and c-jun in ATDC5 cells after loading 32G of hypergravity 1 hour daily for 3 days. (B) Time-course of c-Fos mRNA expression after loading 32G of hypergravity for 0, 30, 60, and 120 min. (C) Intensity-dependent effect of hypergravity on c-Fos mRNA expression in ATDC5 cells after loading 1G, 18.7G, 33.3G, 52.0G, 207.9G, and 467.9G for 60 min. mRNA expression of all the genes were analyzed by real-time PCR and normalized by *Gapdh* and *Rpl13a* expression. The bars represent the mean ± standard error. The results are normalized to the control sample (n = 4). p values were obtained by performing a Student’s test; *p<0.05, **p<0.005 compared with the control sample.

32 G of hypergravity was loaded onto the ATDC5 monolayers after 4 days of subculture. The samples were exposed to hypergravity 1 hour daily for 3 days. As a result, 32 G of hypergravity significantly up-regulated the c-fos mRNA expression (0.48-fold change; p < 0.005) but did not significantly affect that of c-jun (1.19-fold change) (Fig 1A).

Since c-fos is known as an early response gene to both biochemical and mechanical stimuli, we performed a time-course experiment to check whether hypergravity exerts its effects during shorter periods. 32 G of hypergravity were therefore loaded onto ATDC5 monolayers for 30 min, 60 min, and 120 min (Fig 1B); as a result, the expression of c-fos was suppressed by, respectively, 34%, 43% and 16%. The greatest reduction by hypergravity was observed when the samples were subjected to 32 G of hypergravity for 60 min (p<0.05). Next, the effect of varying the intensity of hypergravity was investigated by loading hypergravity for 60 min, at 18.7 G, 33.3 G, 52.0 G, 207.9 G, and 467.9 G, and subsequently measuring c-fos expression by rt-PCR; under those intensities, the expression fold-change of c-fos was respectively 0.48, 0.70, 0.75, 1.39, and 0.79 (Fig 1C). The greatest reduction was observed at 18.7 G (p<0.05). Therefore, the optimized conditions for c-fos down-regulation under hypergravity were 18.7 G for 60 min.

### The change in actin cytoskeleton by hypergravity was involved in the down-regulation of c-fos

**Fig 2:**
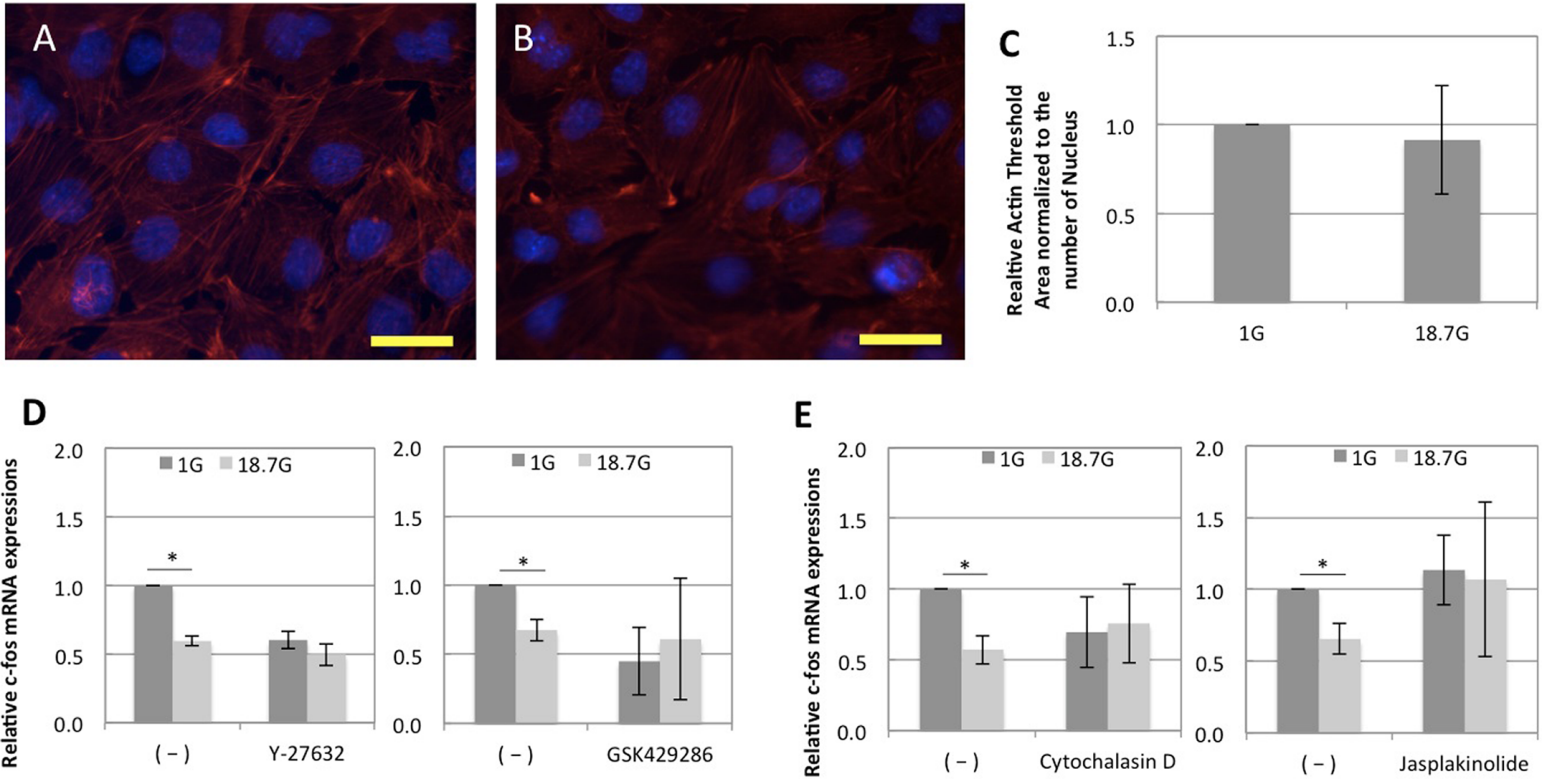
Involvement of actin cytoskeleton in the down-regulation of c-fos by hypergavity. Staining of cell nuclei (with DAPI, in blue) and actin filaments (with rhodamin phalloidin, in red) after loading 18.7G of hypergravity for 60 min; (A) 1G (B) 18.7G. The scale bar represents 50μm.(C) Actin threshold area normalized to the number of nucleus was quantified from the result of Actin immunostaining (n = 4). The results were expressed as relative amounts against the expression of control sample (n = 4). Inhibitor tests: (D) Y27632 and GSK429286 and (E) Cytochalasin D and Jasplakinolide were utilized respectively as a ROCK inhibitor and an actin polymerization inhibitor. c-fos mRNA expression was analyzed by real-time PCR and normalized to *Gapdh* and *Rpl13a* expression. Each value is the average of three experiments. The bars represent the mean ± standard error. p values were obtained by performing a Student’s test; *p<0.05, **p<0.005 compared with the control sample.

Based on the previous results, inhibitor tests were performed under the above optimized conditions (18.7 G for 60 min). First, actin staining was carried out to check any change in the actin filaments after loading 18.7 G of hypergravity for 60 min (Fig 2A and 2B). The staining showed a slight reduction in actin filaments. The bundles of actin filaments observed under control conditions (1 G) became relatively blurred after exposure to hypergravity.

Then, we carried out inhibitor tests utilizing ROCK inhibitors (Y27632 and GSK429286) and actin polymerization inhibitors (Cytochalasin D and Jasplakinolide). By addition of Y27632 and GSK429286 in Fig 2D, the significant down-regulations in c-fos mRNA expression under hypergravity of the negative control samples were non-significantly inhibited to 0.84-fold change and 1.36-fold change, respectively. The relative fold changes in c-fos expression under hypergravity by adding Y27632 and GSK429286 compared to the native control group were 1.52-fold change (p = 0.27) and 2.11-fold change (p = 0.27), respectively. In Fig 2E, Cytochalasin D and Jasplakinolide also non-significantly inhibited the effect of hypergravity on c-fos down-regulations for the negative control sample to 1.10-fold change and 1.36-fold change. The relative fold changes in c-fos expressions by addition of Cytochalasin D and Jasplakinolide compared to the negative control group were 1.36-fold change (p = 0.31) and 1.23-fold change (p = 0.46), respectively.

### The down-regulation of c-fos was inhibited by Wortmanin through suppression of Akt signaling

**Fig 3:**
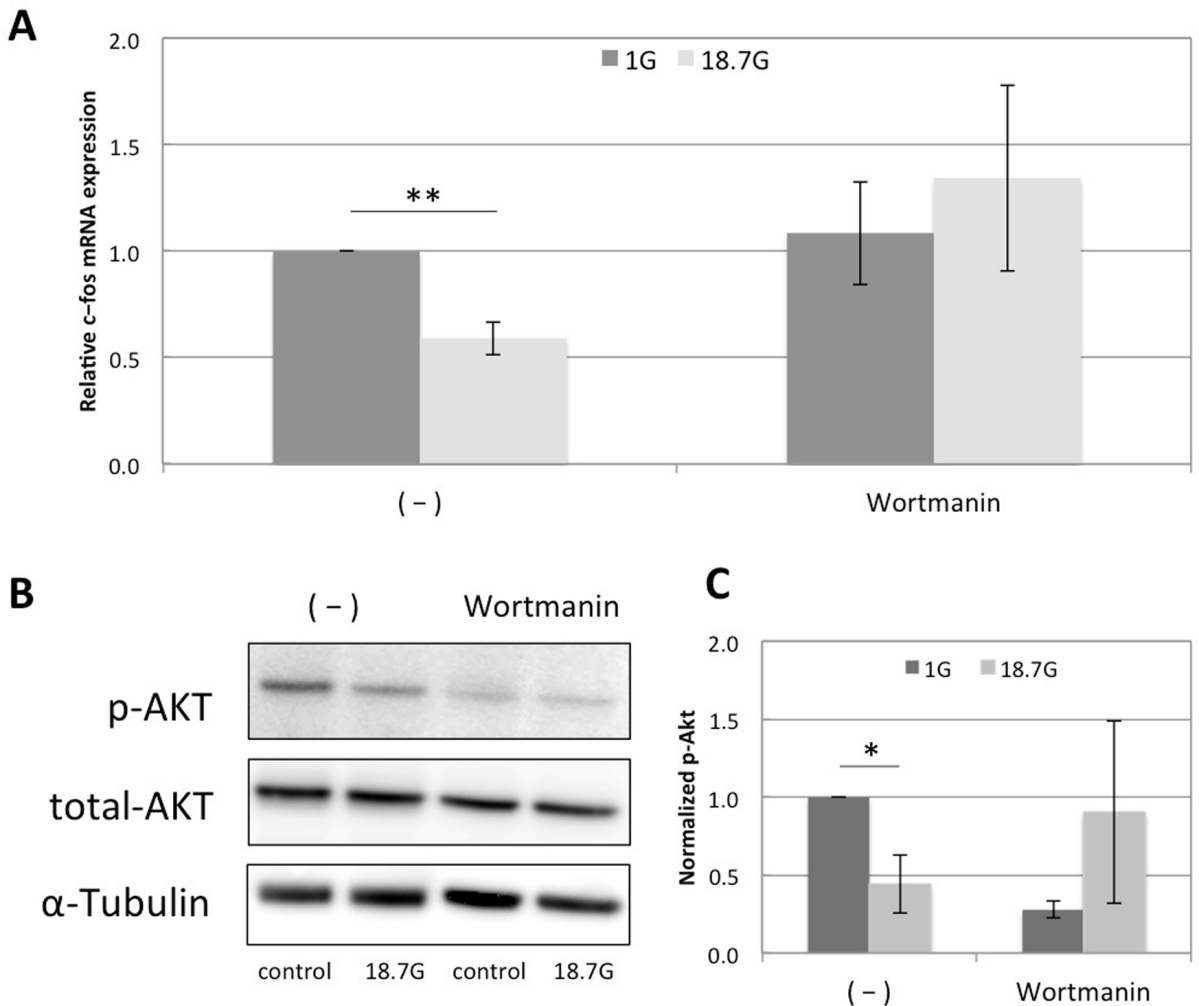
Involvement of PI3K signaling pathway in the down-regulation of c-fos by hypergravity. Wortmanin Inhibitor tests in ATDC5 cells under hypergravity for 60 min. (A) c-fos mRNA expression was analyzed by real-time PCR and normalized to *Gapdh* and *Rpl13a* expression. Wortmanin was utilized as a PI3K inhibitor. (B) Western blotting was carried out to check the change in phosphorylated-Akt (p-Akt), total-Akt, and α-tubulin protein levels after loading 18.7G of hypergravity for 60 min. Wortmanin was added as a PI3K inhibitor to confirm the involvement of the PI3K pathway, particularly Akt. (C) P-Akt levels were normalized to total Akt protein levels. Each value is the average of three experiments. The bars represent the mean ± standard error (n = 3). p values were obtained by performing a Student’s test; *p<0.05, **p<0.005 compared with the control sample.

In order to check the involvement of the PI3K signaling pathway, we performed inhibitor tests using Wortmannin (a PI3K pathway inhibitor). ATDC5 cells were submitted to 18.7 G for 60 min and rt-PCR was carried out. While the down-regulation of c-fos under hypergravity was observed in negative controls, Wortmannin (1.34-fold change in centrifuged samples) significantly inhibited the c-fos down-regulation by hypergravity, as shown in Fig 3A. The fold change between control group and Wortmanin group was 2.58-fold change (p<0.005). Fig 3B presents the results of western blotting for phosphorylated-Akt (p-Akt) and total Akt in the presence or absence of Wortmannin. Quantification of the p-Akt signal normalized to total Akt is shown in Fig 3C. Under hypergravity, Akt phosphorylation was significantly down-regulated (0.44-fold change; p<0.05) while the down-regulation of p-Akt under hypergravity was inhibited by addition of Wortmannin. As the fold change between negative control group and Wortmanin group was 7.41-fold change (p<0.05), the addition of Wortmanin inhibited the down-regulation of Akt phosphorylation against hypergravity.

## Discussion

In this study, we investigated the effect of hypergravity on the murine ATDC5 chondroprogenitor cells. We found no significant change in the mRNA expression of c-jun, which is one member of the AP-1 family that form an AP-1 complex by dimerizing with c-fos[13,31]. We, however, first found that c-fos was significantly down-regulated by hypergravity. This is an opposite phenomenon as other types of mechanical stimuli including hypergravity that are known to up-regulate the expression of c-fos [32]. Our results suggested that the c-fos expression in response to greater hypergravity showed the different manner compared to Fidelina’s study applying 2 G in locus coeruleus cells. Moreover, as an early response gene known to be rapidly modulated by mechanical stimuli such as shear stress, the mRNA expression of c-fos was transiently down-regulated by hypergravity in as little as 30 min. This indicates that c-fos is also an early response and mechano-sensitive transcriptional factor under hypergravity. In other words, the c-fos down-regulation by hypergravity may be a meaningful finding since c-fos is a member of the AP-1 family known to regulate the expression of MMPs, enzymes critically involved in the pathogenesis of OA[15]. Moreover, there is a report that c-fos up-regulation brought about inhibition of chondrocyte differentiation[16]. These results together could have a positive effect on tissue engineering by down-regulating c-fos gene expression.

**Fig 4:**
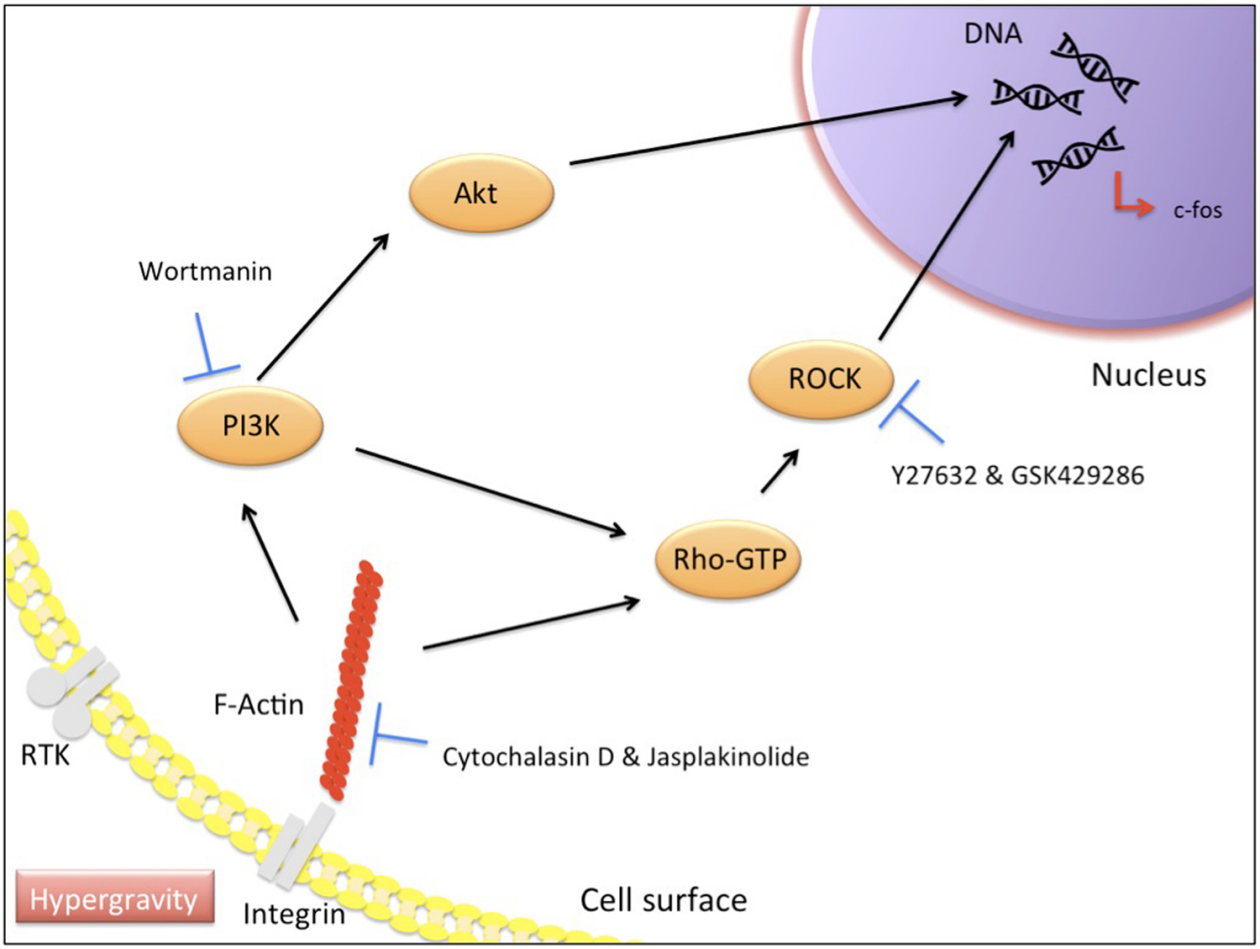
Schematic diagram of signaling pathways involved in c-fos expression in cells under hypergravity.

In order to unravel the mechanism involved, we focused on the actin cytoskeletons in response to hypergravity. Several studies reported that the force generated by actin filaments contributed to cell surface mechanical properties as well as intracellular nuclei[33,34]. As illustrated in Fig 4, we attempted to weaken the actin filaments that connect those nucleus and organelles in the cells by using ROCK inhibitors (Y27632 and GSK429286) or actin polymerization inhibitors (Cytochalasin D and Jasplokinolide). Under hypergravity, a reduction in actin filaments was observed in as little as 1 hour. Moreover, addition of ROCK inhibitors decreased the basal expression of c-fos mRNA in non-centrifuged cells. This may indicate that the basal c-fos expression levels in the cells were maintained by the ROCK/Rho-GTP signaling pathway under a condition of 1 G. In other words, interactions between cell nucleus and the actin cytoskeleton maintained through the ROCK/Rho-GTP signaling pathway under the 1 G condition may have been disconnected under hypergravity, resulting in less tension in cytoskeleton followed by c-fos suppression. As well as the results obtained from actin polymerization inhibitor test, these results altogether imply the involvements of actin cytoskeletons in c-fos gene expressions in the cells. Moreover, c-fos down-regulation by hypergravity may be linked to decreased actin polymerization or tension via the ROCK/Rho-GTP signaling pathway.

On the other hand, we also performed inhibitor tests using Wortmannin to check if the PI3K signaling pathway was involved in the c-fos down-regulation under hypergravity. PI3K is known to be modulated by actin polymerization in mammalian cells[35]. The rt-PCR results showed that the c-fos down-regulation was inhibited by addition of Wortmannin. In order to confirm the involvement of the PI3K signaling pathway, we carried out western blotting to verify the activation of Akt signaling, which is downstream of the PI3K signaling pathway, under hypergravity. We showed that hypergravity suppressed Akt phosphorylation in ATDC5 cells while this down-regulation was inhibited by addition of Wortmannin. Some studies have also reported that inactivation of Akt causes down-regulation of c-fos, preventing osteoclast differentiation [36]. Our findings are consistent with those previous studies. Here, we report that Akt activity is inhibited in chondroprogenitor cells by hypergravity, leading to the down-regulation of c-fos.

The mechanism implicated in the down-regulation of c-fos expression under hypergravity is still not totally understood, but one hypothesis may involve the difference in densities between nucleus and cytoskeleton. Since the density of the cell nucleus (1.4 g/cm^3^) is greater than that of other organelles (1.06 ~ 1.19 g/cm^3^) in cells [37,38], the nucleus may be submitted to stronger pulling forces under hypergravity, inducing changes in the signaling between cell nucleus and cytoskeleton, eventually leading to the down-regulation of c-fos mRNA expression in ATDC5 cells. The reason for the smaller effect on c-fos expression under intensities of hypergravity higher than 18.7 G may be due to the fact that both nucleus and cytoskeleton were pulled down together by excessive hypergravity, resulting in less loss of signaling connections between the nucleus and the cytoskeleton. A real-time observation technique will be required in further studies to observe displacements within the cell under hypergravity.

The major finding of this study is that hypergravity down-regulates c-fos expression in murine ATDC5 chondroprogenitor cells. The inhibition of actin filaments via ROCK/Rho-GTP and PI3K signaling pathways were involved in the c-fos down-regulation under hypergravity. While c-fos induction caused by various mechanical stimuli has been reported, this experimental model using hypergravity showed the opposite phenomenon of c-fos down-regulation. Furthermore, models using hypergravity could suggest a new approach for the field of tissue engineering.

## Abbreviations

AP-1: Active protein 1
MMP-1: Matrix metalloproteinase-1
OA: Osteoarthritis
PI3K: Phosphoinositide 3-kinase
ROCK: Rho-associated, coiled-coil containing protein kinase
